# An Apple Fruit Fermentation (AFF) Treatment Improves the Composition of the Rhizosphere Microbial Community and Growth of Strawberry (*Fragaria × ananassa *Duch ‘Benihoppe’) Seedlings

**DOI:** 10.1371/journal.pone.0164776

**Published:** 2016-10-18

**Authors:** Jie Zhang, Hui Pang, Mengxia Ma, Yufen Bu, Wei Shao, Weijing Huang, Qianlong Ji, Yuncong Yao

**Affiliations:** 1 Department of Plant Science and Technology, Beijing University of Agriculture, Beijing, 102206, China; 2 Beijing Collaborative innovation center for eco-environmental improvement with forestry and fruit trees, Beijing, 102206, China; 3 College of Biological Science and Engineering, Beijing University of Agriculture, Beijing, 102206, China; Wuhan Botanical Garden, CHINA

## Abstract

Plant growth can be promoted by the application of apple fruit fermentation (AFF), despite unclear of the underlying mechanisms, the effects involved in AFF on rhizosphere microorganisms have been hypothesized. We investigated the consequences of applying AFF alone or in combination with *Bacillus licheniformis* to strawberry tissue culture seedlings *in vitro*, the analyses of Denaturing Gradient Gel Electrophoresis (DGGE) and 16S rDNA were performed to determine AFF effects on rhizosphere. Moreover, the growth index and antioxidant enzyme activities were determined 30 days after treatments. We identified five dominant bacteria in AFF: *Coprinus atramentarius*, *Bacillus megaterium*, *Bacillus licheniformis*, *Weissella* and *B*. *subtilis*. The greatest number of bacterial species were observed in the rhizosphere of control matrix (water treated), and the lowest diversity appeared in the rhizosphere soil treated with 10^8^ cfu/mL *B*. *licheniformis* alone. Combining AFF plus *B*. *licheniformis* in one treatment resulted in the largest leaf area, plant height, root length, plant weight, and the markedly higher activities of antioxidant enzymes. We conclude that a combination of AFF plus *B*. *licheniformis* treatment to matrix can increase antioxidant enzymes activities in strawberry seedlings, optimize the status of rhizosphere microbial, and promote plant growth.

## Introduction

Apple fruit fermentation (AFF) is a liquid organic fertilizer that is made from naturally fallen fruits, or those that have detached during crop thinning, by a rapid and simple fermentation method [1]. The AFF process promotes the decomposition of organic matter, releases minerals, biologically active substances and secondary metabolites and enhances the growth of microbes in the soil. AFF can therefore potentially provide nutrients, regulators, and other beneficial compounds to promote and regulate plant growth and development. Moreover, its use can reduce the accumulation of decaying fruit in an orchard environment, and limit soil degradation. Little is known about the mechanisms underlying the effects of AFF on plant growth and development, although a range of other plant waste products, such as cut shoots, drooping flowers, and plant cuttings, are being tested for their ability to similarly enhance plant development and orchard soil quality.

Previous studies have shown that AFF application can elevate plant nutrient contents, promote seedling growth, improve leaf size, increase net leaf photosynthesis and fruit quality of apple (*M*. *pumila* Miller cv. Gongteng Fuji/*M*. *micromalus* Maik), pear (*Pyrus pyrifolia* (Burm.f.). cv. Nakai/*Pyrus aetulaefolia* Aunge) and peach (*Prunus*. *persica* Stoke cv. Dajiubao), presumably due to the addition of nutritionally important factors [2–4]. However, the effects of AFF on the soil of orchard or other field types have not been characterized.

Soils are complex and dynamic systems that undergo a wide range of changes involving physical and chemical factors, mineral elements, root exudates, enzyme systems and soil microorganisms. Of these factors, soil microorganisms are important because changes in their population structure and diversity profoundly influence the decomposition and supply of soil nutrients, enzyme activities and other soil metabolic processes, and affect the structure of the plant community, by modulating plant growth and mediating nutrient cycling [5]. Accordingly, it has been reported that soil microbial diversity is critical for balancing the soil quality, and the rhizosphere represents a critical reservoir of soil microorganisms [6]. Soil microbial communities are affected by plant root exudates and allelopathic chemicals that promote plant growth [7], and microbes in the rhizosphere can, in turn, enhance plant nutrient acquisition [8]. Many studies have shown that plant growth promoting bacteria (PGPB) interact with roots, improving plant growth and health [9–10]. One study showed that genera whose presence in the soil closely correlates with ammonia content are also known for their participation in nitrogen cycling [11].

Some studies have identified specific effects of bacteria on plant physiology and development, *Bacillus amyloliquefaciens* FZB42 stimulates the production of root exudates and promote root growth [12]. Several plant-growth promoting (PGP) bacteria have been shown to enhance plant disease resistance [13] and the presence of bacterial pathogens, such as *Erwinia carotovora* (*Pectobacterium carotovorum*), can cause an increase in the diversity of Alphaproteobacteria and Bacteroidetes and accelerate the natural selection of plant taxa with antimicrobial properties [14–15]. *Bacillus licheniformis* belongs to plant-growth promoting (PGP) bacteria. *Bacillus licheniformis* treated *Arachis hypogea* showed increase in fresh biomass, total length and root length [16]. Other studies have demonstrated the biosorption properties of *Enterobacter sp*. J1, which can take up high levels of the heavy metals Pb, Cd, Cu [17], and *Pseudomonas putida* can absorb Cd, Cu, Pb, Zn [18]. Such processes may provide plants with a more favorable growth environment. However, further studies are needed to establish the effects of *Bacillus licheniformis* on rhizosphere matrix-plant interactions, as well as their spatial-temporal patterns.

A number of enzymes are known to represent physiological indicators of plant stress resistance, including phenylalanine ammonia lyase (PAL), peroxidase (POD) and polyphenol oxidase (PPO), which affect the levels of oxidative compounds. PAL carries out the first committed step in phenylpropanoid metabolism and has been shown to regulate plant growth and disease resistance [19]. The activity of POD has been associated with photosynthesis, respiration and the oxidation of auxin, and its activity also changes during plant growth and development [20]. Finally, PPO activity has been shown to increase significantly upon disease onset in plants [21]. Thus, changes in the activities of these enzymes, in conjunction with growth parameters, can provide an indication of plant resistance to stress.

In this current study we examined the consequences of applying AFF to strawberry (*Fragaria × ananassa* Duch ‘Benihoppe’) seedlings. For successful transplantation, strawberry tissue cultured seedlings must go through an acclimatization period, and their growth and development is dependent on adequate nutrient levels and adaptation to the environment. We investigated whether treating the matrix with AFF during the acclimatization period increased matrix microbial community diversity and ultimately improved the resistance of the seedlings to stress. We performed 16S ribosomal RNA (rRNA) sequencing to identify the dominant bacteria in AFF and the microbial community of the rhizosphere following different AFF treatments, and determined the effects of AFF treatments on the contents of mineral elements, heavy metals, antioxidant enzyme activities in the seedlings, and on seedling growth. The goal was to identify a mechanistic basis for the effects of AFF and a practical framework for AFF application.

## Materials and Methods

### Plant Materials

Naturally abscised/fallen and damaged fruits (*Malus domestica* cv. Red Fuji) were used as the starting material for the fermentation broth. The characteristics of the fruit in the fermentation were as follows: average weight of a single fruit > 150 g, total soluble solids > 12.5%, and titratable acid < 0.6%, with reference Huang. All fruits were collected from the China-Japan Friendly Sightseeing Orchard in the Changping District of Beijing (Public Property; 116.2°E; 40.2°N). For the spraying experiments, strawberry seedlings (*Fragaria × ananassa* Duch ‘Benihoppe’) were transplanted into plugs, which consisted of 50 holes for every seedling tray (specification: top diameter 4.8 cm, bottom diameter 3.0 cm, height < 6.5 cm) with vermiculite:peat (1:1) as the matrix.

### Production of AFF Broth

The AFF broth used in this study was made from the fruit described above and an Oasis 4 enzyme (Jiamusi Lvzhou Company, Jiamusi, Heilongjiang, China) that promote rapid fermentation. All fruits were washed and sterilized with boiling water and then cut into lumps or chopped using a disintegrator. An apple fruit: brown sugar: water: Oasis 4 enzyme mass (500: 20: 200: 10 weight ratio) mixture was placed in a glass bottle, four layers of gauze were used to seal the bottle mouth, and the mixture was stirred for 5 min every 24 h. During the fermentation period the room temperature was maintained at 23–25°C for 30 d, and then the supernatant was transferred to a 4°C refrigerator to be used for the experimental treatments [22].

### Experimental Design

Two days after transplanting, a randomized block of 5 treatments with 3 replications was designed. According to previous studies [22], the treatments were as follows: blank = blank plots with no transplanted seedlings; CK = matrix was sprayed 500 times with sterile deionized water; T1 = matrix was sprayed 500 times with AFF solution; T2 = matrix was sprayed 500 times with AFF solution + 10^8^ cfu/mL *Bacillus licheniformis*; T3 = matrix was sprayed 500 times with 10^8^ cfu/mL *Bacillus licheniformis* in sterile deionized water.

### Determination of Plant Growth Parameters

In the first 30 d after the seedlings had been transferred into plugs, as described above, seedling height, root length, total plant weight, root weight and shoot weight of 30 plants were measured. Loosely adhering matrix was removed from the roots, and the plants were washed with tap water and sterilized deionized water before measuring the growth of the roots and shoots. Fifty healthy leaves from each treatment were collected for leaf weight and leaf area measurements.

### Isolation, Purification and Identification of Dominant Bacterial Strains in AFF

A 0.1 mL volume of AFF was diluted 100 times with 9.9 mL volume of sterilized deionized water then 200 μL of this solution was inoculated into peptone beef extract medium, malt extract agar medium or potato agar medium. After incubation in a 32°C or a 28°C biochemical incubator for 4d, colony morphology was observed and colonies that were growing well were diluted using a spread-plate or streak plate method [1]. The 16S rDNA sequences and ITS sequences of these colonies were then determined as described below.

### DNA Extraction and PCR Amplification of AFF 16S rDNA and (Internal Transcribed Spacer) ITS Sequences

AFF DNA was extracted using a DNA extraction kit (Shanghai Generay Biotech Co., Ltd) according to the manufacturer’s instructions. PCR amplification was performed using the universal ITS sequence primers ITS1 (5’-TCCGTAGGTGAACCTGCG-3’) and ITS4 (5’-TCCTCCGCTTATTGATATGC-3’), and the universal bacterial primers 27F (5’-AGAGTTTGTCCTGGCTCAG-3’) and 1492r (5’- GGTTACCTTGTTACGACTT-3’). ITS PCR was performed using a Bio-Rad T100 thermal cycler (Bio-Rad Laboratories, CA, USA). Amplification was carried out in 50 μL reaction volume with 5.0 μL of 2.5 mM of dNTPs, 2.0 μL of 20 μM ITS1 primer and 2.0 μL of 12.5 μM ITS4 primer, 5.0 μL of 10×buffer (Mg^2+^), 1 μL of ExTaq DNA polymerase, 1 μg template DNA, and variable volume ddH_2_O. The PCR program consisted of an initial denaturation step of 1 min at 94°C, followed by 35 cycles of 30 s at 94°C, 30 s at 55°C and 90 s at 72°C, and a final elongation step for 5 min at 72°C. After resolving the amplicons by agarose gel electrophoresis, those within the appropriate size range were cut from the gel and purified using a Qiaquick gel extraction kit (Shanghai Generay Biotech Co., Ltd), prior to sequencing. PCR amplification of 16S rDNA was performed using a Biometra T-gradient thermal cycler (Bio-Rad Laboratories, CA, USA) in a reaction volume of 50 μL, containing 5.0 μL of 2.5 mM of dNTPs, 2.0 μL of 20 μM 27F primer and 2.0 μL of 12.5 μM 1492r primer, 2.0 μL of 10× buffer (Mg^2+^), 1 μL of ExTaq DNA polymerase, 1 μg template DNA, and variable volume ddH_2_O. The PCR program consisted of an initial denaturation step of 1 min at 94°C, followed by 35 cycles of denaturation at 94°C for 30s, annealing at 58°C for 30s, elongation at 72°C for 90s and a final elongation step at 72°C for 5 min. PCR products were verified on an agarose gel, and amplicons within the appropriate size range were cut from the gel and purified using the Qiaquick gel extraction kit (Shanghai Generay Biotech Co., Ltd) prior to sequencing.

### Matrix DNA Extraction and PCR Amplification of 16S rRNA Gene Fragments

Seedlings were carefully removed from the matrix and root-adhering matrix particles (< 0.02 mm in diameter) were collected with a brush, chilled on ice immediately and then stored at -20°C for subsequent processing. DNA in the matrix samples was extracted using a modified CTAB method according to DNA extraction kit (Tiangen biotech (Beijing), Co. Ltd). The 16S rRNA genes were amplified [23] using the universal bacterial primers GC-338F (5’- CGCCCGGGGCGCGCCCCGGGGCGGGGCGGGGGCGCGGGGGGCCT- ACGGGAGGCAGCAG-3’) and 518R (5’- ATTACCGCGGCTGCTGG-3’) with the addition of a barcoding sequence. 16S rRNA amplification PCR reactions were performed using a Biometra T-gradient thermal cycler in a reaction volume of 50 μL, containing 3.2 μL of 2.5 mM dNTPs, 2.0 μL of 20 mM GC-338 F primer and 2.0 μL of 20 mM 518 R primer, 5.0 μL of 10× buffer, 0.4 μL rTaq, 50 ng template DNA, and variable volume ddH_2_O. Before amplification, an initial denaturation step of 94°C for 5 min was performed, followed by 30 cycles of 94°C for 1 min, 55°C for 45 s, and 72°C for 1 min, and. a final elongation step at 72°C for 10 min. After resolving the amplicons by agarose gel electrophoresis, amplicons within the appropriate size range were cut from the gel and PCR products were purified using a DNA Gel Extraction Kit (Omega Bio-Tek) following the manufacturer’s protocol, prior to sequencing.

### Denaturing Gradient Gel Electrophoresis (DGGE) Analysis of PCR Products

10 μL of PCR product subjected to DGGE analysis [24], using a gradient of 35–55% for 5 h at 150 V and 60°C. To obtain a clear image, the gel was photographed with the gel photo system (Gel-Doc2000, Bio-Rad, USA), which identifies the bands occupying the same position in the different lanes of the gel. Adopted OMEGA Company Poly-Gel DNA Extraction Kit recycling target band, and the sequencing was performed by HuaDa Genomics Institute. Searches in BLAST from GenBank were used to find the closest known relatives to the partial 16S rDNA sequences. Sequences with 97% were considered to represent the same species.

### Determination of the Content of Mineral Elements and Heavy Metals in Seedling Leaves

Leaves from the seedlings grown in matrix subjected to the different treatments for 30 d were collected to measure the contents of mineral elements and heavy metals. Samples were dried at 105°C for 3–5 h, and element analysis was performed using Kjeldahl determination and inductively coupled plasma mass spectrometry (ICP-MS) as previously described [25–26].

### Determination of POD, PAL and PPO Activities in the Seedling Leaves

Samples of leaves from seedlings grown in matrix subjected to the different treatments for 2–3 days or 30 d were collected for enzyme activity determination at 0 h, 3 h, 6 h, 9 h and 12 h after treatment. The POD, PAL and PPO activities were determined using the POD, PAL and PPO kit (Suzhou Comin Biotechnology Co., Ltd).

### Statistical Analysis

We analyzed the dominant bacterial species of the AFF broth itself, as well as the broth that had been supplemented with *B*. *licheniformis*. The BLAST program in GenBank [27] was used to identify the 16S rDNA sequences. The banding patterns of DGGE profiles were analyzed by Quantity One software (Bio-Rad). A Neighbor-joining phylogenetic tree was constructed using the MEGA5 software [24], the diversity of the microbial community was estimated using the Shannon diversity index (H), and evenness and richness (S) [28]. All data were subjected to a one-way analysis of variance (ANOVA) using SPSS and the treatment means were compared using the Duncan’s multiple range test with p < 0.05. Each data point was the mean of 3 replicates and was expressed as mean ± standard error (SE).

## Results

### Molecular Identification of Dominant Strains in the AFF

**Fig 1:**
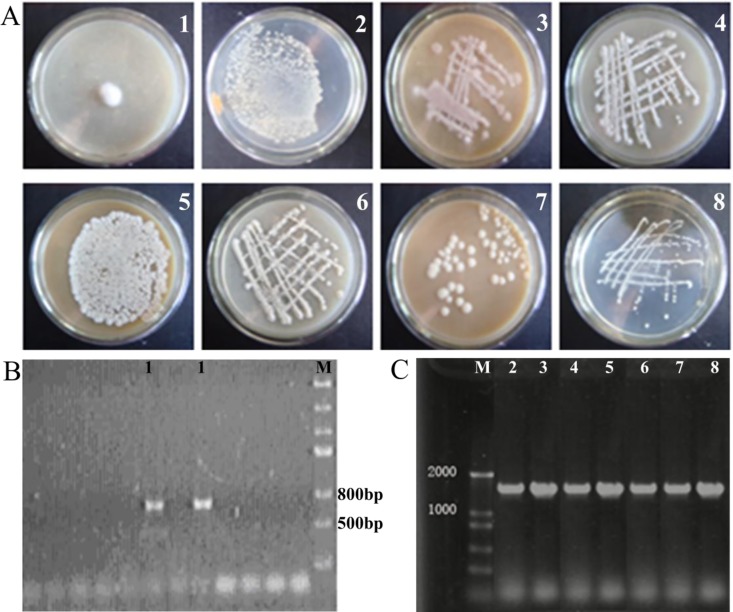
Colony morphology of the dominant strains in the culture medium and electrophoresis atlas of the PCR products amplified from apple fruit fermentation (AFF). (A), colony morphology. 1, *Coprinus atramentarius* (Bull.) *Fr*. 2, *Bacillus megaterium*. 3, *Bacillus licheniformis*. 4, *Weissella*. 5–8, *Bacillus subtilis*. (B, C), PCR amplification from apple fruit fermentation (AFF).

In order to identify the dominant strains of the microbial community in AFF, the supernatant was first used to inoculate beef extract peptone and malt extract agar medium. We identified eight different strains dividing on the medium based on differences in colony. Specifically, they exhibited white smooth moist surfaces, a mucoid appearance with pink wrinkles, two types with white sags, white wrinkles and rough surfaces, circular protrusions, or white opaque wrinkles. All of the eight strains had regular circular edges (Fig 1A). Specific PCR primers were then used to amplify microbial 16S rDNA (bacteria) or ITS (fungus) fragments (Fig 1B and 1C, S1 Table), and the dominant bacteria were shown to be *Coprinus atramentarius*, *Bacillus megaterium*, *B*. *licheniformis* and *Weissella*. These correspond to the type 1 to 4 morphology described above, and the bacteria with morphology type 5–8 were identified as *B*. *subtilis* (Fig 1A). The results revealed the presence of many kinds of bacteria and fungi in the AFF. Therefore, we hypothesized that *B*. *megaterium*, *B*. *licheniformis* and *B*. *subtilis* may regulate the soil microbial community and improve plant growth.

### Characterization of the Rhizosphere Microbial Community under Various Treatments

**Fig 2:**
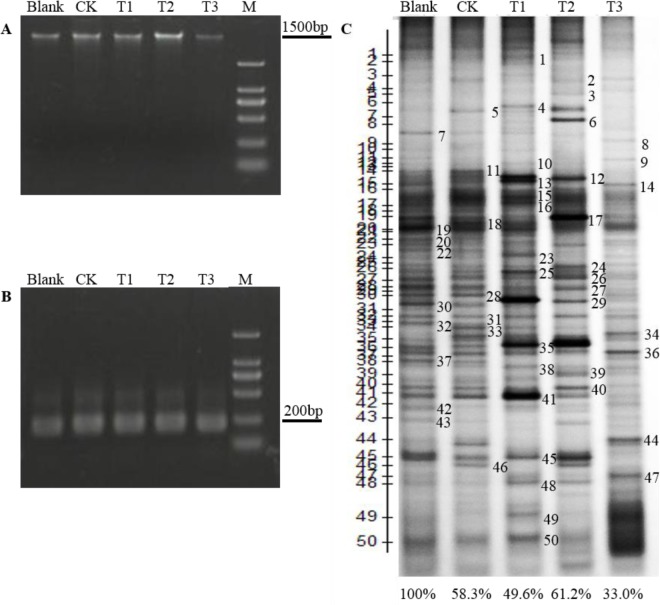
The identification of the rhizosphere microbial community under various treatments. (A), agarose gel (2%) electrophoresis of rhizosphere genomic DNA (Marker: DL2000). (B), agarose gel (2%) electrophoresis of PCR amplified 16S rDNA (Marker: DL2000). (C), PCR-DGGE (denaturing gradient gel electrophoresis) DNA profiles, and the sequencing codes for cloning of bands that differed between treatments. Abbreviation: Blank (blank plots transplanting the plant before), CK (500 mL water), T1 (apple fruit fermentation (AFF) 500 times solution), T2 (AFF 500 times solution + 10^8^ cfu/mL *Bacillus licheniformis*), T3 (10^8^ cfu/mL *Bacillus licheniformis*).

To investigate the rhizosphere microbial community after applying AFF and/or bacterial solution (500 times solution + 10^8^ cfu/mL *Bacillus licheniformis*) to the matrix, 5 experimental treatments were designed, as described in Materials and Methods, and PCR analysis of 16S rDNA fragments in the matrix samples was conducted using DGGE (Fig 2A and 2B). The DGGE fingerprint chromatogram (Fig 2), in which each band represents a different bacterium, showed substantial differences in the number of bands, band intensities, and locations of the microorganism DNA from different areas around the strawberry rhizosphere. Compared with the blank and CK treatments, the number of bacteria in T1 increased in bands 6 and 8, and the intensities of bands 13, 15, 16, 25, 35, 41 and 45 were significantly higher; the number of bacteria in T2 was higher in bands 3 and 5, and the intensities of bands 12 and 29 were significantly greater in T3; the number of bacteria was lower in bands 2, 8 and 5 compared with the blank, T1 and T2, and the intensities of all bands were lower than those in other treatments. These results suggested that application of AFF to the matrix resulted in a greater microbial species diversity and abundance around the strawberry rhizosphere, these effects were even greater by adding *B*. *licheniformis* to the AFF.

### The Similarity and Diversity of Bacterial Community Structures under Various Treatments

To determine the microbial community structure in the strawberry rhizosphere, we performed a calculation of the pairwise similarity coefficient based on the sequencing results of the DGGE analysis, and a clustering diagram was created using UPGMA (S1 Fig, S2 Table). The similarity coefficient of the microorganism population structures in the strawberry rhizosphere of T1 to blank and CK were 50% and 52%, and those of T1 to T2 and T3 were 56.7% and 29.2%, respectively. The similarity coefficient of T2 to the blank, CK and T3 were 61.2%, 52.6% and 29.1%, respectively, while the similarity coefficient of T3 to the blank and CK were 33% and 42.4%, respectively (Table 1). These results indicated the differences in the microorganism population structures under various treatments.

**Table 1:** The similarity of the rhizosphere microbial community under various treatments.

Treatments	Blank	CK	T1	T2	T3
Blank	100	58.3	50	61.2	33
CK	—	100	52	52.6	42.4
T1	—	—	100	56.7	29.2
T2	—	—	—	100	29.1
T3	—	—	—	—	100

The diversity of microbial metabolic functions was inferred by the richness and diversity index, and Shannon diversity index was used to assess the species richness, where the value has a positive correlation with the diversity of metabolic functions. A Shannon-Wiener analysis of strawberry rhizosphere microorganisms indicated the highest value for the CK treatment, followed by the blank, T1 and T3, and the lowest value for T2. The Richness analysis values for strawberry rhizosphere microorganism populations showed the same relative order (Table 2). The Evenness analysis of strawberry rhizosphere microorganisms gave the highest value for the CK treatment, followed by the Blank, T1 and T2, and the lowest value for T3. These analyses suggested that AFF plus *Bacillus licheniformis* treatment increased the abundance of functional bacterial groups, and maintained the evenness in soil microbial community, although the Shannon-wiener index and richness decreased.

**Table 2:** The diversity of the rhizosphere microbial community under various treatments.

Treatments	Shannon-Wiener	Evenness	Richness
Blank	3.43	0.99	32
CK	3.51	0.99	35
T1	3.33	0.98	30
T2	3.12	0.98	26
T3	3.23	0.97	28

### Strawberry Seedling Growth under Various Treatments

**Fig 3:**
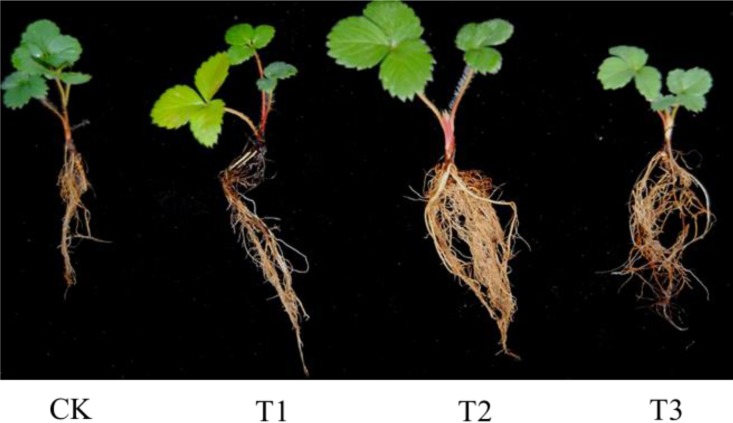
The growth of strawberry seedlings treated with apple fruit fermentation (AFF) plus *Bacillus licheniformis* after 30 d. (A), the morphological characteristics of the strawberry seedlings. Abbreviation: CK (500 mL water), T1 (AFF 500 times solution), T2 (AFF 500 times solution + 10^8^ cfu/mL *Bacillus licheniformis*), T3 (10^8^ cfu/mL *Bacillus licheniformis*).

To investigate the effect of the different treatments on strawberry growth, the characters of seedlings were analyzed as shown in Fig 3. The results showed that the T2 treatment not only significantly increased the number of roots (Fig 3), root length and root weight, but also increased the plant height, leaf area, leaf weight, and plant weight compared to the CK treatment (Table 3). Compared with T1 or T3 treatment, the seedlings treated by T2 also displayed increased trends in the growth status. We concluded that the combination of AFF plus *B*. *licheniformis* promoted root and plant growth, as evidenced by an increased accumulation of biomass in strawberry seedlings.

**Table 3:** The growth status of the strawberry seedlings treated with with AFF plus *Bacillus licheniformis* after 30 d.

Parameters	CK	T1	T2	T3
Plant height (cm)	4.5±0.29b	4.87±0.18ab	5.4±0.31a	4.97±0.15ab
Leaf area(cm^2^)	2.24±0.06b	3.11±0.18a	3.32±0.16a	2.90±0.07a
Root length (cm)	6.77±0.15b	11±0.58a	11±0.58a	10±0.58a
Plant Weight (g)	0.66±0.04c	0.78±0.03b	0.96±0.03a	0.89±0.03a
Root Weight (g)	0.28±0.02c	0.017±0.02c	0.49±0.03a	0.41±0.02b
Leaf and stem Weight (g)	0.39±0.18b	0.46±0.02a	0.48±0.03a	0.45±0.02ab

Lowercase letters indicated significance of P<0.05 by Duncan’s multiple range test.

### Mineral Element Contents in Leaves under Various Treatments

To investigate the mechanisms by which the AFF plus *B*. *licheniformis* promoted plant growth, we measured the mineral element contents in leaves of the seedlings, and observed substantial differences as a consequence of the different treatments. T2 leaves had the highest levels of phosphorous (P), potassium (K), iron (Fe), magnesium (Mg) and boron (B), while for the nitrogen (N) content in leaves showed CK>T1>T2>T3. The relative copper (Cu) contents were T3>T2>CK>T1, and the zinc (Zn) levels were T3>T2>T1>CK. The manganese (Mn) content in the leaves was lower for the T2 and T3 treatments than for the other treatments. For the heavy metal elements, T1 had a lower mercury (Hg) content compared with the other treatments, and T1, T2 and T3 had lower lead (Pb) levels. T1 and T2 had lower chromium (Cr) contents than the CK treatment, while T3 had higher cadmium (Cd) content but no differences in Hg and Cr compared to CK (Table 4). From these data we concluded that spraying AFF plus *B*. *licheniformis* significantly increased the contents of a range of elements, reduced the contents of heavy metals, and improved the growth conditions of the strawberry seedlings.

**Table 4:** The effect of AFF plus *Bacillus licheniformis* on mineral element and heavy metal contents in the leaves of strawberry seedlings.

Elements(mg/Kg)	CK	T1	T2	T3
N (%)	3.42±0.06a	3.30±0.03ab	3.19±0.09b	2.95±0.07c
P	671.27±6.15c	642.07±5.58d	888.43±9.15a	752.7±10.81b
K	3700.67±25.57d	4060.33±27.12c	4342.15±42.15a	4200±16.80b
Fe	98.62±1.39c	146.2±4.41b	166.83±5.48a	104.52±3.04c
Cu	2.34±0.04b	1.29±0.03c	2.35±0.05b	2.54±0.04a
Mn	20.53±0.46b	25.13±0.77a	17±0.33c	18.17±0.54c
Zn	7.69±0.07d	8.48±0.19c	9.37±0.04b	9.82±0.05a
Mg	928.47±16.10c	1066.33±35.31b	1202.67±8.37a	1190±40.41a
B	311.67±2.19d	342.8±2.10c	385±3.48a	375.8±2.84b
Hg	0.17±0.01a	0.09±0.01b	0.16±0.01a	0.18±0.01a
Pb	0.93±0.04a	0.16±0.02b	0.01±0.003c	0.02±0.04c
Cr	2.49±0.08a	2.1±0.09b	2.19±0.03b	2.46±0.08a
Cd	0.03±0.003b	0.04±0.005b	0.01±0.006b	0.05±0.003a

Lowercase letters indicated significance of P<0.05 by Duncan’s multiple range test.

### Effects of AFF plus *B*. *licheniformis* on the POD, PAL and PPO Activities of Strawberry Seedlings

**Fig 4:**
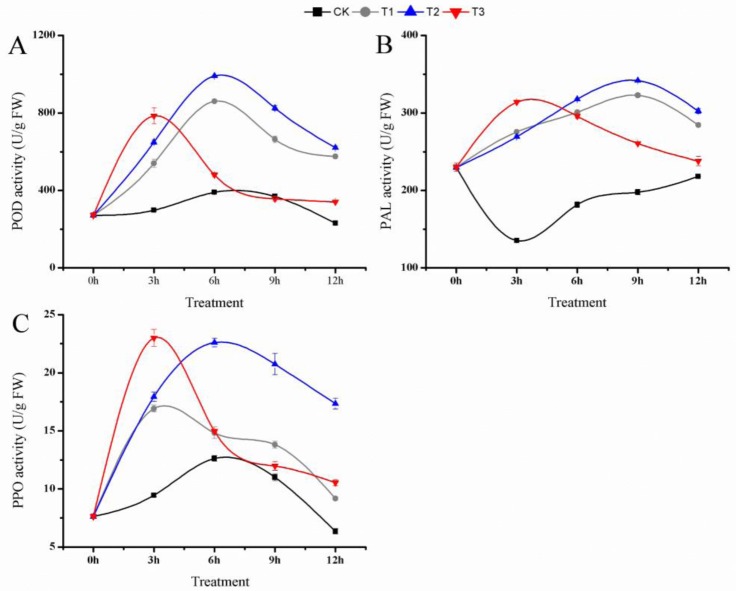
Change of activities of peroxidase (POD), phenylalanine ammonia lyase (PAL) and polyphenol oxidase (PPO) after applying apple fruit fermentation (AFF) plus *Bacillus licheniformis*. (A, B, C), the activities of POD, PAL, PPO after applying AFF plus *Bacillus licheniformis* for 12 h. CK (500 mL water), T1 (AFF 500 times solution), T2 (AFF 500 times solution + 10^8^ cfu/mL *Bacillus licheniformis*), T3 (10^8^ cfu/mL *Bacillus licheniformis*). Vertical bars represent mean values and error bars indicate ±SE (n = 3).

In order to determine the effects of the AFF plus *B*. *licheniformis* treatment on stress resistance in strawberry seedlings, we measured the antioxidant index, a value based on POD, PAL and PPO activities, 3 d and 30 d after transplanting. We observed that POD activity first increased and then decreased during the course of the treatment, and that in general the activities in the T2, T1 and T3 samples were significant higher than in the CK sample. Activities in the T3 leaves and in the T2 leaves reached the highest levels at 6 h (Fig 4A). PAL activity in the different treated plants emerged varying degrees rose. While the activity in the CK sample decreased after treatment for 3 h and then increased for the remainder of the treatment time, the activities in the T2, T3 and T1 samples were significant higher than the CK sample during the entire treatment period, and the activity in the T2 samples reached a maximum at 9 h (Fig 4B). PPO activity following different treatments showed an initial increase and then decreased during the course of the treatment, and the PPO activity in T2 sample was significantly higher than in other sample after treatment for 4.5 h, and the T2 PPO activity reached a maximum at 6 h and remained at a high level (Fig 4C). After treatments for 30 days, the POD, PPO and PAL activities were higher in the T2 leaves than in other treatments (Table 5). These results suggested that applying AFF plus *B*. *licheniformis* to the matrix significantly promoted the resistance and survival rate of the transplants, leading to a higher content of partial elements and improved seedling growth.

**Table 5:** Changes in the activities in strawberry leaves of POD, PAL and PPO after applying AFF plus *Bacillus licheniformis* for 30 d.

Enzyme activity(U/g FW)	CK	T1	T2	T3
POD	139.18±3.57d	344.61±10.95c	744.24±10.68a	475.67±10.84b
PAL	252.98±0.59d	314.40±0.06b	330.54±0.51a	273.51±0.1c
PPO	2.27±0.13c	4.2±0.42b	10.4±0.23a	3.87±0.27b

Lowercase letters indicated significance of P<0.05 by Duncan’s multiple range test.

### Redundancy Analysis of Matrix Microbial Parameters, Element Content and Enzymatic Activities under Various Treatments

**Fig 5:**
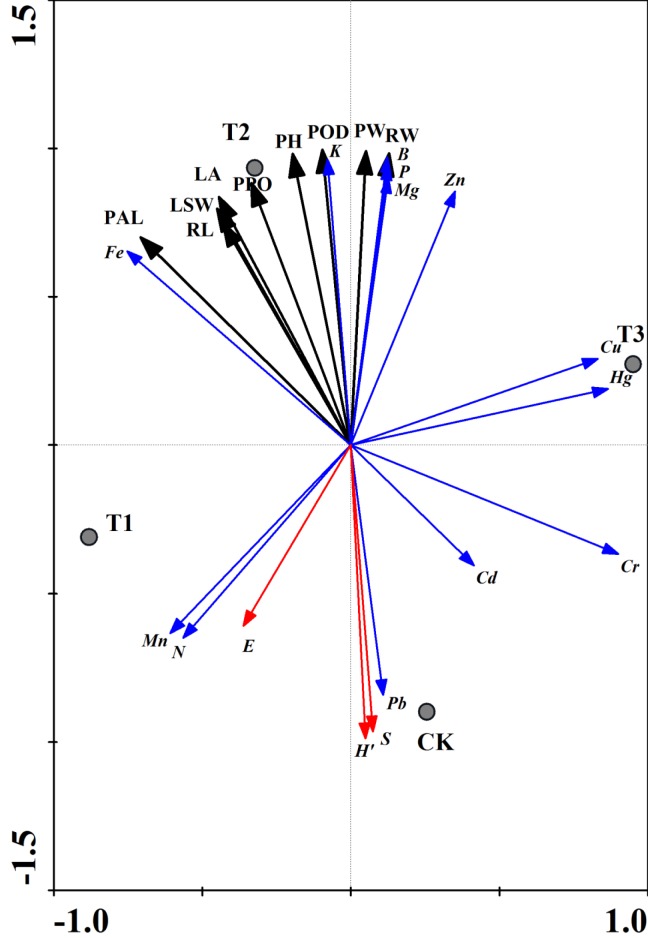
Redundancy analysis (RDA) plot based on the rhizosphere microbial diversity with the plant growth index and antioxidant enzyme activity under different treatments. PH, Plant height; LA, Leaf area; RL, Root length; PW, Plant Weight; RW, Root Weight; LSW, Leaf and stem Weight. H′, Shannon-Wiener index; E, evenness index; S, richness. The blue lines represent mineral element content. The red lines show microbial diversity indices. The black lines indicate plant growth index and antioxidant enzyme activity.

To identify associations between strawberry rhizosphere microorganism populations, element contents and enzymatic activities, we performed redundancy analysis (RDA) to investigate relationships with matrix microbial parameters, element contents and enzymatic activities variables (e.g., N, P, K, POD). Plant height, leaf area, root length, plant weight, root weight, leaf and stem weight and POD, PAL, PPO activities were found to be greatly increased by the T2 treatment, while elevated levels of Fe, B, Mg and Zn also significantly correlated with the T2 treatment. Matrix microbial diversity was also enhanced by the CK treatment, and Shannon-Wiener index (H′), richness (S), evenness index (E), and N and Mg significantly correlated with T1 treatment. However, the H′, S and E were reduced by the T2 treatment, and Cu, Hg, Cr and Cd levels were negatively corrected to T2 samples, reaching values nearer to those of the T3 and CK samples. Significant separation trends were observed between different treatments based on an RDA diagram (Fig 5).

## Discussion

### Mechanism of AFF Promote Plant Growth

Many crops, particularly horticulturally important plants and transplanted trees, require a relatively long rejuvenation period, and plants propagated through plant tissue culture even more. During this recovery period, young seedlings are sensitive to soil, climate and environmental factors for their survival, growth and development, and the promotion of seedling growth through this period and a reduced recovery time have been the focus of many horticulturists and silviculturists. Nutrient supplying is undoubtedly an important aspect of seedling recovery and in this regard there is interest in developing simple liquid organic fertilizers, such as AFF, which consists of fermented naturally dropped apple fruit and fruit collected by artificial crop thinning, as well as from other residual plant material from horticultural production. Two application approaches have been used: foliar sprays or application to the matrix in order to improve the matrix nutrient environment and promote seedling growth and resistance. However, a better understanding of the impact of these fermentation liquids on soil factors and the subsequent growth of transplanted seedlings is important to enhance their practical application.

Apple fruit fermentation (AFF) contains relatively abundant mineral elements, bioactive substances and secondary metabolites and a supplemented microbial population. As a simple liquid organic fertilizer, spraying AFF onto fruit trees has been reported to improve growth conditions and promoted fruit quality [29], and also to inhibit plant infection with harmful bacteria due to its weak acid properties. These outcomes are similar to those that have shown with foliar fertilizer application. In apple, Liu *et al* [29] found that the application of AFF significantly promoted the growth and increased the photosynthetic efficiency of young and adult apple trees, and Chen *et al* [30] confirmed the beneficial effects of nutrient solutions on the physiological activities of apple trees and fruit quality. Furthermore, adding *Alternaria alternata* f. sp. *mali*. to apple and chitosan fermentation broth significantly enhanced superoxide dismutase and catalase activities, causing a decrease in the content of malondialdehyde and the accumulation of reactive oxygen species, along with increased plant stress resistance [31].

### AFF Influence the Rhizosphere Microbial Community

Soil microbial systems are influenced by complex interactions between soil nutrient contents, soil enzymes, heavy metals and the physical and chemical properties. Diversity in the soil microbial community has been shown to be crucial for integrated soil fertility improvement [32]. The addition of AFF, with high levels of C and N and kinds of microbial species, influences soil composition, structure and the diversity of the microbial community, especially increases the abundance of dominant bacterial population, thereby improving the soil nutrient profiles [33]. The application of chemical fertilizers, such as N, P and K, increased nutrient availability for the plant and induced a change in the microbial community structure, which in turn promoted plant growth [34]. Meanwhile, the addition of N to soil decreased the abundance of *Verrucmicrobia*, while there is a close relationship between soil N availability and associated functional genes, which in turn shaped the soil microbial composition [35–36]. Donnison *et al* [37] compared the effect of traditionally and intensively management submontane haymeadows on the soil microbial community, found the aboveground plant community diversity decreased, and the soil microbial community size and composition weakened, but did not affect the functional capability of the soil microbial community with respect to soil ecosystem-level functions of organic matter decomposition and nutrient recycling. This was because the single microbial function diversity makes up for the function inadequate caused by soil microbial diversity decrease. Our results indicated that nutrient solution plus *bacillus licheniformis* affected the composition of microbial community in cultural soil, resulting in the increasing the abundance of the dominant physiological bacterial community, with that, the diversity index and richness of microbial community were reduced slightly. This may be due to the interaction among 5 kinds of the dominant physiological bacterial population including plus *bacillus licheniformis* in the spraying solution. Studies have shown that most of the creatures to maintain the ecological system function may be redundant [38]. Under stable conditions, only a few populations in maintaining ecosystem functioning is necessary, but when the environment disturbance, most of the population has a role to maintain the ecosystem balance, referred to as the insurance hypothesis [39]. It was noted that the blank was closer to T2 than CK according to the composition of bacterial in clustering results based on the unweighted pair group method using the Arithmetic Average, one of main reason may be that spraying water significantly changes the composition of soil microorganism community, another reason may come from the balance effect of interaction of the dominant biological population in spraying mixture solution on the composition of soil microorganism community. Meanwhile, the interaction of plants on the ground to soil microorganism was included in these changes. These were important for further researches of agricultural practices to soil microbial community.

The results of the present study indicated that treating the matrix with AFF plus *B*. *licheniformis* significantly improved the number of matrix bacterial species, composition, but decreased the diversity indices, compared with other treatments. This was associated with the presence of particularly dominant strains, such as *Coprinus atramentarius*, *B*. *megaterium*, *B*. *subtilis*, W*eissella* and *B*.*licheniformis* in the fermentation process. *Coprinus atramentarius*, has been reported to secrete organic acids and other active substances that act as antibacterial agents [40]. In *Camellia sinensis*, adding *B*. *megaterium* promoted the tea plant growth, which maybe stimulates solubilization of phosphate and the production of Indole Acetic Acid (IAA), siderophores and antifungal metabolites [41]. The promotion of plant growth and reduction in disease intensity have been shown to be due to a combination of mechanisms. *B*. *subtilis* can modify the interactions between soil bacteria, and the *B*. *subtilis* can produce a variety of antimicrobial substances, most of which are polypeptides, including iturins, surfactins, bacillonmycins, and mycosubtilins [42]. Strawberry root rot disease, caused by *Fusarium oxysporum*, was markedly reduced by application of *B*. *subtilis* to the soil, and *B*. *licheniformis* can produce a variety of antimicrobial substances, high temperature proteases and a range of growth factors that inhibit the growth of pathogenic bacteria and promote the proliferation of beneficial microorganisms [43–44]. We observed that applying AFF plus *B*. *licheniformis* to the matrix not only provided substantial amounts of mineral elements to the strawberry seedlings, but also increased the number of bacterial species and the diversity of the bacterial community, thereby providing a better environment for the transplanted seedlings.

### Mechanism of AFF Promote Plant Resistance

The antioxidant enzyme activities and heavy metal contents of seedlings provide indications of their stress resistance status. In this study we found that the application of AFF and AFF plus *B*. *licheniformis* increased antioxidant activities in seedling leaves, and reduced the heavy metal contents. This correlated with increases in the length of the plants and roots, leaf area and dry matter accumulation, plant weight, root weight, and stem and leaf weights. We infer from these results that the AFF and AFF plus *B*. *licheniformis* treatments enhance the antioxidant capacity but decrease the heavy metal pollution of the seedlings, and that these resulted from an increase in the diversity of the soil bacterial community, the supply of soil nutrients, and the enhancement of soil chemistry properties. Microbes in the soil rhizosphere assist with nutrient acquisition and promote plant growth, potentially through fixing N, dissolving minerals of the soil and increasing plant absorption of moisture [8]. Moreover, dead *B*. *licheniformis* cells can absorb heavy metals from the environment, thereby decreasing heavy metal contents [45], and bacteria are capable of directly or indirectly altering strawberry root auxin levels and changing the root architecture, thereby promoting the growth of strawberry tissue culture seedlings [46].

### Application Prospects of Plant Material Fermentation Broth

**Fig 6:**
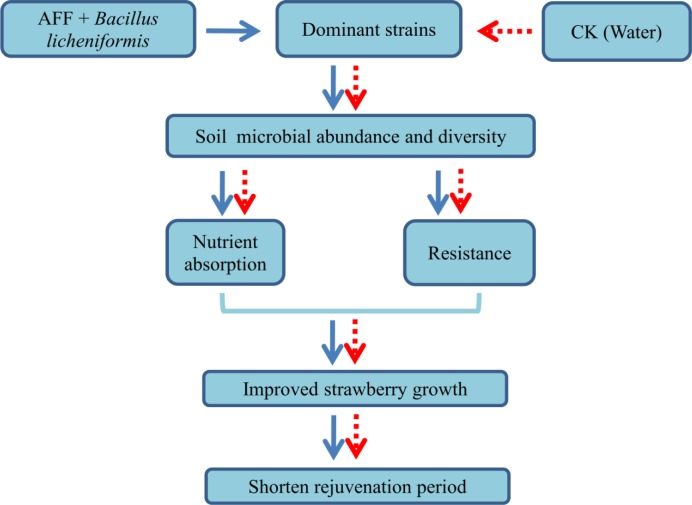
Schematic representation of the effect of spraying apple fruit fermentation (AFF) plus *Bacillus licheniformis* on promoting plant growth.

We propose that AFF plus *B*. *licheniformis* can enhance microbial composition, structure, and diversity, promote the absorption of nutrients by seedlings, and increase stress resistance, thereby promoting seedling survival and vigor during the rejuvenation period (Fig 6), which is consistent with results in *Arabidopsis* [13]. Given the different waste materials and fermentation conditions used, the nutritional quality, the types of dominant bacteria and the different biologically active substances in the AFF produced can differ. This would be expected to have varying effects on the soil is different, but be generally beneficial for soil microbes and their interactions with plants. During the fermentation process, in order to improve the nutrient deficiencies, poor performance, invasion by specific pathogens and other issues, chitin, iron, *B*. *subtilis*, and *B*. *licheniformis* or other components can be added [31, 33, 47]. Further research to identify such factors and optimize their usage, together with the optimization of fermentation material and different bacteria, will lead to the wider use of this technology.

## Supporting Information

S1 Fig Clustering based on the unweighted pair group method using the Arithmetric Average for 5 Treatments. Abbreviations: Treatments: Blank (blank plots transplanting the plant before), CK (500 mL water), T1 (apple fruit fermentation (AFF) 500 times solution), T2 (AFF 500 times solution + 10^8^ cfu/mL *Bacillus licheniformis*), T3 (10^8^ cfu/mL *Bacillus licheniformis*).
(TIF)

S2 Fig Phylogenetic tree constructed using the Neighbor-Joining method. (TIF)

S1 Table The 16S and 18S rDNA sequences of the dominant strains microbial in apple fruit fermentation (AFF). (DOCX)

S2 Table Sequencing results of the Denaturing Gradient Gel Electrophoresis (DGGE) bands. (DOCX)
