# Supplementary figures and images for: An Apple Fruit Fermentation (AFF) Treatment Improves the Composition of the Rhizosphere Microbial Community and Growth of Strawberry (Fragaria × ananassa Duch ‘Benihoppe’) Seedlings
Source: PLoS One. 2016 Oct 18;11(10):e0164776. doi: 10.1371/journal.pone.0164776 (PMC5068704; doi:10.1371/journal.pone.0164776)

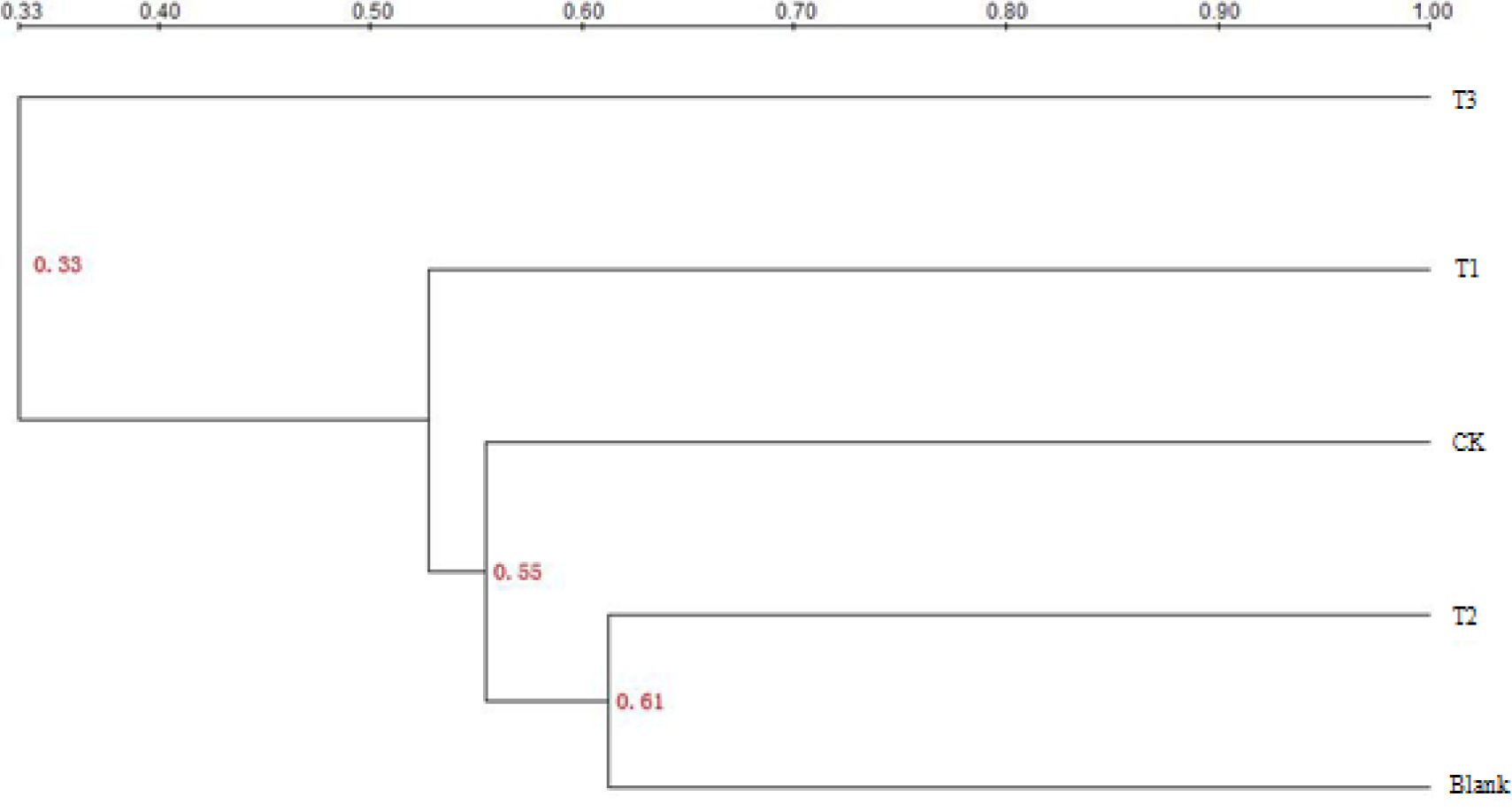

Supplement: S1 Fig — Abbreviations: Treatments: Blank (blank plots transplanting the plant before), CK (500 mL water), T1 (apple fruit fermentation (AFF) 500 times solution), T2 (AFF 500 times solution + 108 cfu/mL Bacillus licheniformis), T3 (108 cfu/mL Bacillus licheniformis). (TIF) [file pone.0164776.s001.tif]

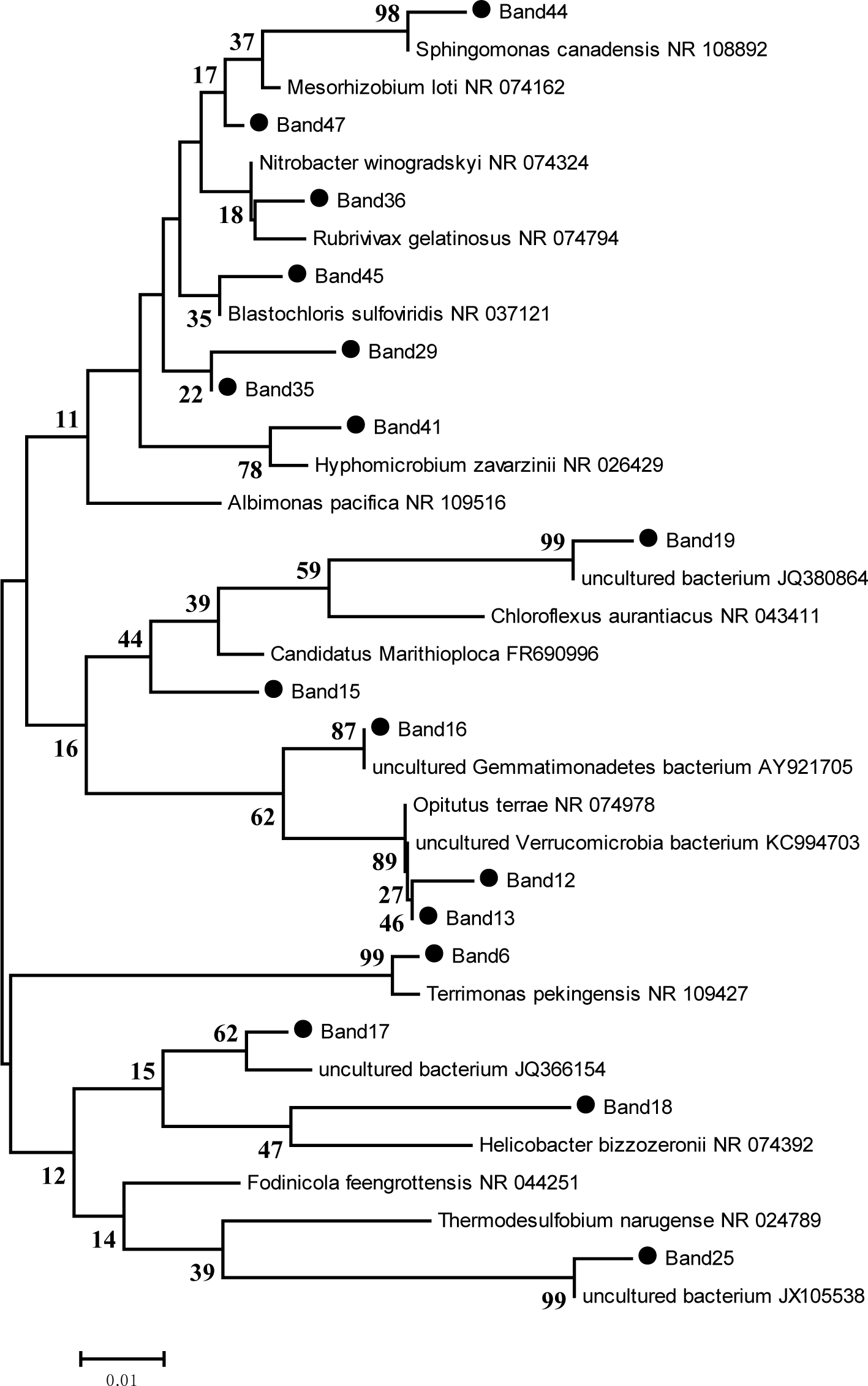

Supplement: S2 Fig — (TIF) [file pone.0164776.s002.tif]
