# Supplementary material for: An Apple Fruit Fermentation (AFF) Treatment Improves the Composition of the Rhizosphere Microbial Community and Growth of Strawberry (Fragaria × ananassa Duch ‘Benihoppe’) Seedlings
Source: PLoS One. 2016 Oct 18;11(10):e0164776. doi: 10.1371/journal.pone.0164776 (PMC5068704; doi:10.1371/journal.pone.0164776)
Supplement: S1 Table — (DOCX) [file pone.0164776.s003.docx]

S1 Table. The 16S and 18S rDNA sequences of the dominant strains microbial in apple fruit fermentation (AFF).

| **Number** | **Sequence** |
| --- | --- |
| **1** | 1 CGTGCACATGCTCCAGAGCAGCTACAACCAAGACCCATAATTTTTCTTAAAGATCCTCTT  61 CCGTAAGGTGAACCTGCGGAAGGATCATTAACGAATAACTATGGTGTCTTGGTTGTAGCT  121 GGCTCCTCGGAGCATTGTGCACGCCCGCCATTTTTATCTATCCACCTGTGCACCGACTGT  181 AGGTCTGGATGACTCTCGTGCTCTCTGAGTGCGGATGCGAGGATTGCCCTTCAACTCGGA  241 GGTGTCTCTCCTCGAATTTCCAGGCTCTACGTCTTTTTACACACCCCAAAAGCATGATAT  301 AGAATGTAGTCAATGGGCTTGATCGCCTATAAAACACTATACAACTTTCAGCAACGGATC  361 TCTTGGCTCTCGCATCGATGAAGAACGCAGCGAAATGCGATAAGTAATGTGAATTGCAGA  421 ATTCAGTGAATCATCGAATCTTTGAACGCACCTTGCGCTCCTTGGTATTCCGAGGAGCAT  481 GCCTGTTTGAGTGTCATTAAATTCTCAACCTCACCCGTTTTCTGAACGGTTCTCCGAGGC  541 TTGGATGTGGGGGTTTGTGCAGGCTGCCTCAGCGCGGTCCGCTCCCCTGAAATGCATTAG  601 CGAGTTCGTACTGAGCTCCGTCTATTGGTGTGATAATTATCTACGCCGTGGACAGGGTTT  661 AGACTCGCTTCTAACCGTCCGCAAGGACAATACCTTTTGACAATTTGACCTCAAATCAGG  721 TAGGACTACCCGCTGAACTTAAGCATATCAATAAGCGGAGGAAGGATCATTAAGAATAAC  781 TATGGTGTCTTGGTTGTGCGGCTCTCGGAGCATTG |
| **2** | 1 GCTATCATGCAAGTCGAGCGACTGATTAGAAGCTTGCTTCTATGACGTTAGCGGCGGACG  61 GGTGAGTAACACGTGGGCAACCTGCCTGTAAGACTGGGATAACTTCGGGAAACCGAAGCT  121 AATACCGGATAGGATCTTCTCCTTCATGGGAGATGATTGAAAGATGGTTTCGGCTATCAC  181 TTACAGATGGGCCCGCGGTGCATTAGCTAGTTGGTGAGGTAACGGCTCACCAAGGCAACG  241 ATGCATAGCCGACCTGAGAGGGTGATCGGCCACACTGGGACTGAGACACGGCCCAGACTC  301 CTACGGGAGGCAGCAGTAGGGAATCTTCCGCAATGGACGAAAGTCTGACGGAGCAACGCC  361 GCGTGAGTGATGAAGGCTTTCGGGTCGTAAAACTCTGTTGTTAGGGAAGAACAAGTACGA  421 GAGTAACTGCTCGTACCTTGACGGTACCTAACCAGAAAGCCACGGCTAACTACGTGCCAG  481 CAGCCGCGGTAATACGTAGGTGGCAAGCGTTATCCGGAATTATTGGGCGTAAAGCGCGCG  541 CAGGCGGTTTCTTAAGTCTGATGTGAAAGCCCACGGCTCAACCGTGGAGGGTCATTGGAA  601 ACTGGGGAACTTGAGTGCAGAAGAGAAAAGCGGAATTCCACGTGTAGCGGTGAAATGCGT  661 AGAGATGTGGAGGAACACCAGTGGCGAAGGCGGCTTTTTGGTCTGTAACTGACGCTGAGG  721 CGCGAAAGCGTGGGGAGCAAACAGGATTAGATACCCTGGTAGTCCACGCCGTAAACGATG  781 AGTGCTAAGTGTTAGAGGGTTTCCGCCCTTTAGTGCTGCAGCTAACGCATTAAGCACTCC  841 GCCTGGGGAGTACGGTCGCAAGACTGAACTCAAGGAATTGACGGGGGCCCGCACAAGCGG  901 TGGAGCATGTGGTTTAATTTCGAAGCCACGCGAGATCTTACCAAGGTCTTGACATCCTCT  961 GACACTCTAGAGATAGAGCGTCCCTCGGGGACGAGTGACAGTGTGCATGATGTCGTCAGC  1021 TCGTGTCCGTGAGATGTAGCTAAGTCCGCAACGAGGGCATCCATGATCTAGTTGCAGCAT  1081 CAGTGGGACTCTACGTGACTGTCGT |
| **3** | 1 GCTATCATGCAAGTCGAGCGGACGACGGGAGCTTGCTCCCTTAGGTCAGCGGCGGACGGG  61 TGAGTAACACGTGGGTAACCTGCCTGTAAGACTGGGATAACTCCGGGAAACCGGGGCTAA  121 TACCGGATGCTTGATTGAACCGCATGGTTCAATTATAAAAGGTGGCTTTTAGCTACCACT  181 TACAGATGGACCCGCGGCGCATTAGCTAGTTGGTGAGGTAACGGCTCACCAAGGCGACGA  241 TGCGTAGCCGACCTGAGAGGGTGATCGGCCACACTGGGACTGAGACACGGCCCAGACTCC  301 TACGGGAGGCAGCAGTAGGGAATCTTCCGCAATGGACGAAAGTCTGACGGAGCAACGCCG  361 CGTGAGTGATGAAGGTTTTCGGATCGTAAAACTCTGTTGTTAGGGAAGAACAAGTACCGT  421 TCGAATAGGGCGGTACCTTGACGGTACCTAACCAGAAAGCCACGGCTAACTACGTGCCAG  481 CAGCCGCGGTAATACGTAGGTGGCAAGCGTTGTCCGGAATTATTGGGCGTAAAGCGCGCG  541 CAGGCGGTTTCTTAAGTCTGATGTGAAAGCCCCCGGCTCAACCGGGGAGGGTCATTGGAA  601 ACTGGGGAACTTGAGTGCAGAAGAGGAGAGTGGAATTCCACGTGTAGCGGTGAAATGCGT  661 AGAGATGTGGAGGAACACCAGTGGCGAAGGCGACTCTCTGGTCTGTAACTGACGCTGAGG  721 CGCGAAAGCGTGGGGAGCGAACAGGATTAGATACCCTGGTAGTCCACGCCGTAAACGATG  781 AGTGCTAAGTGTTAGAGGGTTTCCGCCCTTTAGTGCTGCAGCAAACGCATTAAGCACTCC  841 GGCCTGGGGAGTACGGTCGCAAGACTGAAACTCAAGGAATTGACGGGGGCCCGCACAAGC  901 GGTGGAGCATGTGGTTTAATTCGAAGCACGCGAGATCTTAACAGTCTTGACATCCTCTGA  961 CACCTAGAGATAGGCCTTCCCCTTCGGGGCCAGATGACAGTTGTTGCATGCTGTCGTCAG  1021 CTCAGTCTGAATGTAGGATAAGTCGCACGAGCGCAACTATGACTAGCTGCAGCATCAGTG  1081 CACTTACGGGTACTGCGGGTTCACCAGAACGT |
| **4** | 1 GCTATCATGCAGTCGACGCCTTGTCGTTTTAATGAATGGAGTGCTTGCACGATATGATTT  61 AAAACAATGCAAGGAGTGGCGAACGGGTGAGTAACACGTGGGAAACCTACCTCTTAGCAG  121 GGGATAACATTTGGAAACAGATGCTAATACCGTATAATACTAAAAACCGCATGGTTTTTA  181 TTTGAAAGATGGTTCTGCTATCACTAAGAGATGGTCCCGCGGTGCATTAGTTAGATGGTG  241 AGGTAATGGCTCACCATGACGATGATGCATAGCCGAGTTGAGAGACTGATCGGCCACAAT  301 GGGACTGAGACACGGCCCATACTCCTACGGGAGGCAGCAGTAGGGAATCTTCCACAATGG  361 GCGCAAGCCTGATGGAGCAACGCCGCGTGTGTGATGAAGGGTTTCGGCTCGTAAAGCACT  421 GTTGTAAGAGAAGAATGTACTTGAGAGTAACTGTTCAAGTAGTGACGGTATCTTACCAGA  481 AAGGGACGGCTAAATACGTGCCAGCAGCCGCGGTAATACGTATGTCCCAAGCGTTATCCG  541 GATTTATTGGGCGTAAAGCGAGCGCAGACGGTTATTTAAGTCTGAAGTGAAAGCCCTCAG  601 CTCAACTGAGGAATTGCTTTGGAAACTGGATGACTTGAGTGCAGTAGAGGAAAGTGGAAC  661 TCCATGTGTAGCGGTGAAATGCGTAGATATATGGAAGAACACCAGTGGCGAAGGCGGCTT  721 TCTGGACTGTAACTGACGTTGAGGCTCGAAAGTGTGGGTAGCAAACAGGATTAGATACCC  781 TGGTAGTCCACACCGTAAACGATGAGTGCTAGCTGTTCGAGGGTTTCCCGCCCTTGAGTG  841 GCGTAGCTAACGCATTAAGCACTCCGCCTGGGGAGTACGACCGCAAGGTTGAAACTCAAG  901 GATTGACGGGGACCGCACAGCGGTGGAGCATGTGTTTATCGAGCACGCGAGACTACAGTC  961 CTGGACATCTTGACACTCAGAGATGGAGCTTCCCTCCGGACAAGTGACAGTGTGCATGAT  1021 GTCGTCAGCTCAGTCTGAATGTAGGTAAGTTCGACGAGCTTCAATGTAGACAGCATCAGT  1081 GGTCCTCATTAGCTGA |
| **5** | 1 GCTATCATGCAAGTCGAGCGGAAGATGGGAGCTTGCTCCCTGATGTTAGCGGCGGACGGG  61 TGAGTAACACGTGGGTAACCTGCCTGTAAGACTGGGATAACTCCGGGAAACCGGGGCTAA  121 TACCGGATGCTTGTTTGAACCGCATGGTTCAGACATAAAAGGTGGCTTCGGCTACCACTT  181 ACAGATGGACCCGCGGCGCATTAGCTAGTTGGTGAGGTAACGGCTCACCAAGGCGACGAT  241 GCGTAGCCGACCTGAGAGGGTGATCGGCCACACTGGGACTGAGACACGGCCCAGACTCCT  301 ACGGGAGGCAGCAGTAGGGAATCTTCCGCAATGGACGAAAGTCTGACGGAGCAACGCCGC  361 GTGAGTGATGAAGGTTTTCGGATCGTAAAGCTCTGTTGTTAGGGAAGAACAAGTGCCGTT  421 CAAATAGGGCGGCACCTTGACGGTACCTAACCAGAAAGCCACGGCTAACTACGTGCCAGC  481 AGCCGCGGTAATACGTAGGTGGCAAGCGTTGTCCGGAATTATTGGGCGTAAAGGGCTCGC  541 AGGCGGTTTCTTAAGTCTGATGTGAAAGCCCCCGGCTCAACCGGGGAGGGTCATTGGAAA  601 CTGGGGAACTTGAGTGCAGAAGAGGAGAGTGGAATTCCACGTGTAGCGGTGAAATGCGTA  661 GAGATGTGGAGGAACACCAGTGGCGAAGGCGACTCTCTGGTCTGTAACTGACGCTGAGGA  721 GCGAAAGCGTGGGGAGCGAACAGGATTAGATACCCTGGTAGTCCACGCCGTAAACGATGA  781 GTGCTAAGTGTTAGGGGGTTTCCGCCCCTTAGTGCTGCAGCTAACGCATTAAGCACTCCG  841 CCTGGGGAGTACGGTCGCAAGACTGAAACTCAAGGAATTGACGGGGGCCCGCACAAGCGG  901 TGGAGCATGTGGTTTATTCGAAGCAACGCGAGAACCTTACCAGGTCTTGACATCCTCTGA  961 CAATCTAGAGATAGGACGTCCCCTTCGGGGGCAGGTTGACAGTTGTGCATGGATGTCGTC  1021 AGCTCGTGTCGTGAGATGGTGGATTAAGTCCGCAACGGAGGCAGCCATGATTACTTGCAG  1081 CATCAGTGGTCGCTTTACGGTACTGCCGGGCGAACACAACG |
| **6** | 1 GCTATCATGCAAGTCGAGCGGAAGATGGGAGCTTGCTCCCTGATGTTAGCGGCGGACGGG  61 TGAGTAACACGTGGGTAACCTGCCTGTAAGACTGGGATAACTCCGGGAAACCGGGGCTAA  121 TACCGGATGCTTGTTTGAACCGCATGGTTCAGACATAAAAGGTGGCTTCGGCTACCACTT  181 ACAGATGGACCCGCGGCGCATTAGCTAGTTGGTGAGGTAACGGCTCACCAAGGCGACGAT  241 GCGTAGCCGACCTGAGAGGGTGATCGGCCACACTGGGACTGAGACACGGCCCAGACTCCT  301 ACGGGAGGCAGCAGTAGGGAATCTTCCGCAATGGACGAAAGTCTGACGGAGCAACGCCGC  361 GTGAGTGATGAAGGTTTTCGGATCGTAAAGCTCTGTTGTTAGGGAAGAACAAGTGCCGTT  421 CAAATAGGGCGGCACCTTGACGGTACCTAACCAGAAAGCCACGGCTAACTACGTGCCAGC  481 AGCCGCGGTAATACGTAGGTGGCAAGCGTTGTCCGGAATTATTGGGCGTAAAGGGCTCGC  541 AGGCGGTTTCTTAAGTCTGATGTGAAAGCCCCCGGCTCAACCGGGGAGGGTCATTGGAAA  601 CTGGGGAACTTGAGTGCAGAAGAGGAGAGTGGAATTCCACGTGTAGCGGTGAAATGCGTA  661 GAGATGTGGAGGAACACCAGTGGCGAAGGCGACTCTCTGGTCTGTAACTGACGCTGAGGA  721 GCGAAAGCGTGGGGAGCGAACAGGATTAGATACCCTGGTAGTCCACGCCGTAAACGATGA  781 GTGCTAAGTGTTAGGGGGTTTCCGCCCCTTAGTGCTGCAGCTAACGCATTAAGCACTCCG  841 CCTGGGGAGTACGGTCGCAAGACTGAAACTCAAGGAATTGACGGGGGCCCGCACAGCGGT  901 GAGCATGTGGTTTAATTCGAAGCAACGCGAGAACCTTACCAGGTCTTGACATCCTCTGAC  961 ATCCTAGAGATAGACGTCCCCTTTCGGGGCAGAGTGACAGTGTGCATGATTGTCGTCAGC  1021 TCGTTCAGGAATGTGGGTTAAGTCCGCACGAGCCAGCCATGACTAGTTGCAGCATCAGTG  1081 CACCTCTTAGATACTTGCGGTGTGTACTCACACCGTTGA |
| **7** | 1 GCGCGCTATCATGCAAGTCGAGCGGAAGATGGGAGCTTGCTCCCTGATGTTAGCGGCGGA  61 CGGGTGAGTAACACGTGGGTAACCTGCCTGTAAGACTGGGATAACTCCGGGAAACCGGGG  121 CTAATACCGGATGGTTGTTTGAACCGCATGGTTCAGACATAAAAGGTGGCTTCGGCTACC  181 ACTTACAGATGGACCCGCGGCGCATTAGCTAGTTGGTGAGGTAACGGCTCACCAAGGCGA  241 CGATGCGTAGCCGACCTGAGAGGGTGATCGGCCACACTGGGACTGAGACACGGCCCAGAC  301 TCCTACGGGAGGCAGCAGTAGGGAATCTTCCGCAATGGACGAAAGTCTGACGGAGCAACG  361 CCGCGTGAGTGATGAAGGTTTTCGGATCGTAAAGCTCTGTTGTTAGGGAAGAACAAGTGC  421 CGTTCAAATAGGGCGGCACCTTGACGGTACCTAACCAGAAAGCCACGGCTAACTACGTGC  481 CAGCAGCCGCGGTAATACGTAGGTGGCAAGCGTTGTCCGGAATTATTGGGCGTAAAGGGC  541 TCGCAGGCGGTTTCTTAAGTCTGATGTGAAAGCCCCCGGCTCAACCGGGGAGGGTCATTG  601 GAAACTGGGGAACTTGAGTGCAGAAGAGGAGAGTGGAATTCCACGTGTAGCGGTGAAATG  661 CGTAGAGATGTGGAGGAACACCAGTGGCGAAGGCGACTCTCTGGTCTGTAACTGACGCTG  721 AGGAGCGAAAGCGTGGGGAGCGAACAGGATTAGATACCCTGGTAGTCCACGCCGTAAACG  781 ATGAGTGCTAAGTGTTAGGGGGTTTCCGCCCCTTAGTGCTGCAGCTAACGCATTAAGCAC  841 TCCGCCTGGGGAGTACGGTCGCAAGACTGAAACTCAAAGGAATTGACGGGGGCCCGCACA  901 AGCGGTGGAGCATGTGATTATTCGAAGCAACGCGAAGAATCTTACCAGTCTTGACATCTC  961 TGACCATCTTAGAGATAGACGTCCCCTCCGGGGCCAGAGTGACAGTTGTGCATGCTGTCG  1021 TCAGCTCGTGTCCGGAATGTGGATTAAGTCCGCACGAAGCGCACCTTGATTAGTTGCCAG  1081 ACTCAGTGGCACCTTAAGGTACTGCGGTGAC |
| **8** | 1 CGGGCTATCATGCAAGTCGAGCGGAAGATGGGAGCTTGCTCCCTGATGTTAGCGGCGGAC  61 GGGTGAGTAACACGTGGGTAACCTGCCTGTAAGACTGGGATAACTCCGGGAAACCGGGGC  121 TAATACCGGATGGTTGTTTGAACCGCATGGTTCAGACATAAAAGGTGGCTTCGGCTACCA  181 CTTACAGATGGACCCGCGGCGCATTAGCTAGTTGGTGAGGTAACGGCTCACCAAGGCGAC  241 GATGCGTAGCCGACCTGAGAGGGTGATCGGCCACACTGGGACTGAGACACGGCCCAGACT  301 CCTACGGGAGGCAGCAGTAGGGAATCTTCCGCAATGGACGAAAGTCTGACGGAGCAACGC  361 CGCGTGAGTGATGAAGGTTTTCGGATCGTAAAGCTCTGTTGTTAGGGAAGAACAAGTGCC  421 GTTCAAATAGGGCGGCACCTTGACGGTACCTAACCAGAAAGCCACGGCTAACTACGTGCC  481 AGCAGCCGCGGTAATACGTAGGTGGCAAGCGTTGTCCGGAATTATTGGGCGTAAAGGGCT  541 CGCAGGCGGTTTCTTAAGTCTGATGTGAAAGCCCCCGGCTCAACCGGGGAGGGTCATTGG  601 AAACTGGGGAACTTGAGTGCAGAAGAGGAGAGTGGAATTCCACGTGTAGCGGTGAAATGC  661 GTAGAGATGTGGAGGAACACCAGTGGCGAAGGCGACTCTCTGGTCTGTAACTGACGCTGA  721 GGAGCGAAAGCGTGGGGAGCGAACAGGATTAGATACCCTGGTAGTCCACGCCGTAAACGA  781 TGAGTGCTAAGTGTTAGGGGGTTTCCGCCCCTTAGTGCTGCAGCTAACGCATTAAGCACT  841 CCGCCTGGGGAGTACGGTCGCAAGACTGAACTCAAAGGAATTGACGGGGGCCCGCACAAG  901 CGGTGGAGCATGTGGTTTAATTCGAAGCAACGCGAAGAACCTTACCAGGTCTTGACATCC  961 TCTGACATCTAGAGATAGGAACGTCCCTTCGGGGCAGATGACAGGTGTGCATGACTGTCG  1021 TCAGCTCGGTCTGAATGGTGGATAAGTCCGCAACGAGCGCAACCATGATCTAGTTGCAGG  1081 CATCAGTGGCACTTAGGTGACTGCGGGTCACCAGAATCG |
