# Supplementary material for: An Apple Fruit Fermentation (AFF) Treatment Improves the Composition of the Rhizosphere Microbial Community and Growth of Strawberry (Fragaria × ananassa Duch ‘Benihoppe’) Seedlings
Source: PLoS One. 2016 Oct 18;11(10):e0164776. doi: 10.1371/journal.pone.0164776 (PMC5068704; doi:10.1371/journal.pone.0164776)
Supplement: S2 Table — (DOCX) [file pone.0164776.s004.docx]

S2 Table. Sequencing results of the Denaturing Gradient Gel Electrophoresis (DGGE) bands.

| Band name | Closest relative bacterial strain | Access number | Similarity/% | Phylum |
| --- | --- | --- | --- | --- |
| 6 | *Terrimonaspekingensis* | NR_109427 | 98 | Bacteroidetes |
| 12 | *Opitutus terrae* | NR_074978 | 95 | Verrucomicrobia |
| 13 | *Opitutus terrae* | NR_074978 | 97 | Verrucomicrobia |
| 15 | *CandidatusMarithioploca* | FR690996 | 90 | Proteobacteria |
| 16 | Uncultured Gemmatimonadetes bacterium | AY921705 | 98 | Gemmatimonadetes |
| 17 | *Helicobacter bizzozeronii* | NR_074392 | 86 | Proteobacteria |
| 18 | *Fodinicolafeengrottensis* | NR_044251 | 90 | Actinobacteria |
| 19 | *Chloroflexusaurantiacus* | NR_043411 | 87 | Chloroflexi |
| 25 | *Thermodesulfobiumnarugense* | NR_024789 | 84 | Firmicutes |
| 29 | *Blastochlorissulfoviridis* | NR_037121 | 96 | Proteobacteria |
| 35 | *Rubrivivaxgelatinosus* | NR_074794 | 97 | Proteobacteria |
| 36 | *Nitrobacterwinogradskyi* | NR_074324 | 99 | Proteobacteria |
| 41 | *Hyphomicrobiumzavarzinii* | NR_026429 | 96 | Proteobacteria |
| 44 | *Sphingomonascanadensis* | NR_108892 | 99 | Proteobacteria |
| 45 | *Albimonaspacifica* | NR_109516 | 96 | Proteobacteria |
| 47 | *Mesorhizobiumloti* | NR_074162 | 98 | Proteobacteria |
